# Supplementary figures and images for: Tris inhibits a GH1 β-glucosidase by a linear mixed inhibition mechanism
Source: PLoS One. 2025 Mar 25;20(3):e0320120. doi: 10.1371/journal.pone.0320120 (PMC11936226; doi:10.1371/journal.pone.0320120)

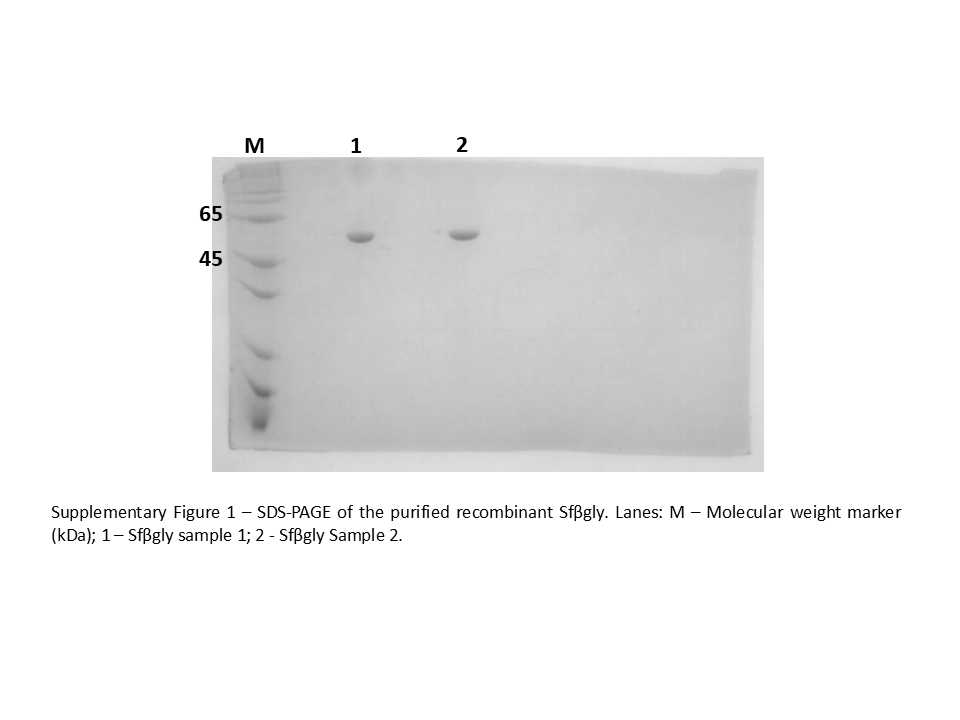

Supplement: S1 Fig — (TIF) [file pone.0320120.s001.tif]

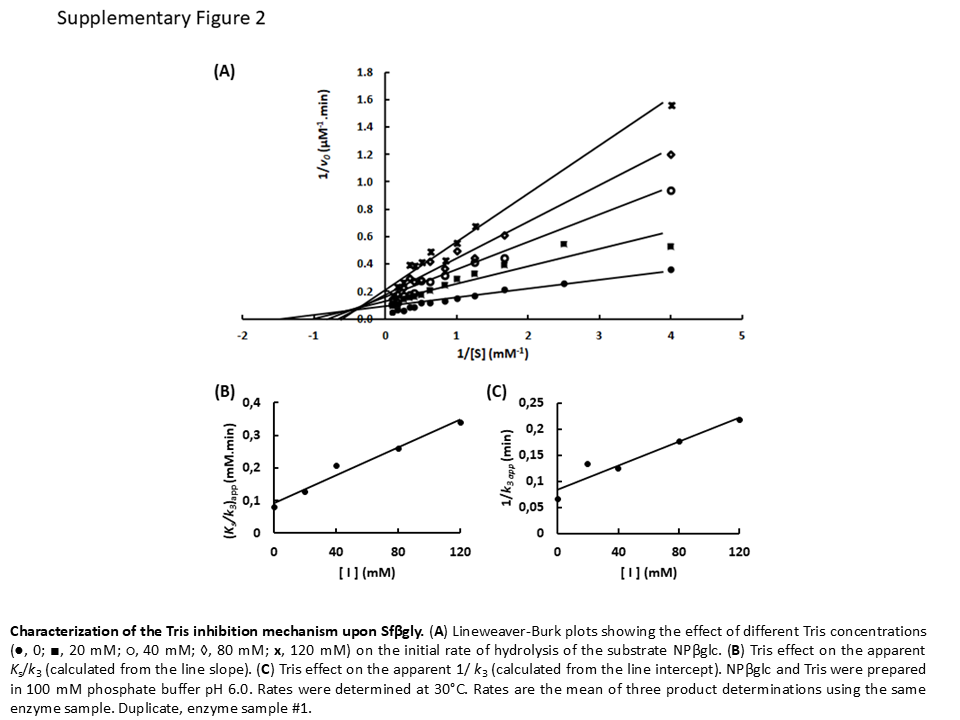

Supplement: S2 Fig — Enzyme sample #1. Substrate NPβglc. (TIF) [file pone.0320120.s002.tif]

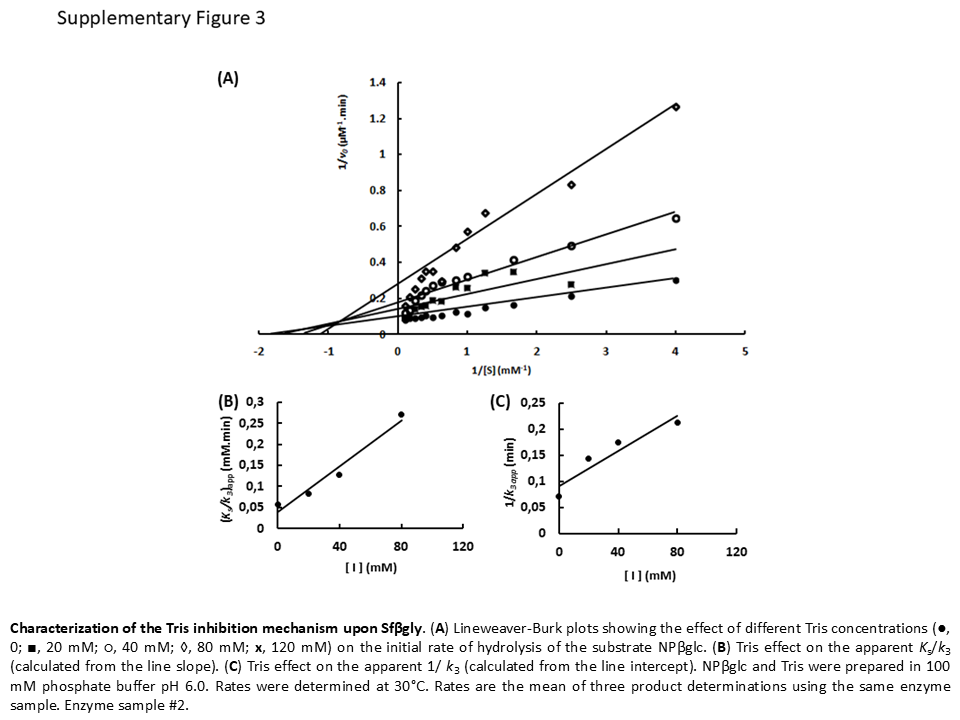

Supplement: S3 Fig — Enzyme sample #2. Substrate NPβglc. (TIF) [file pone.0320120.s003.tif]

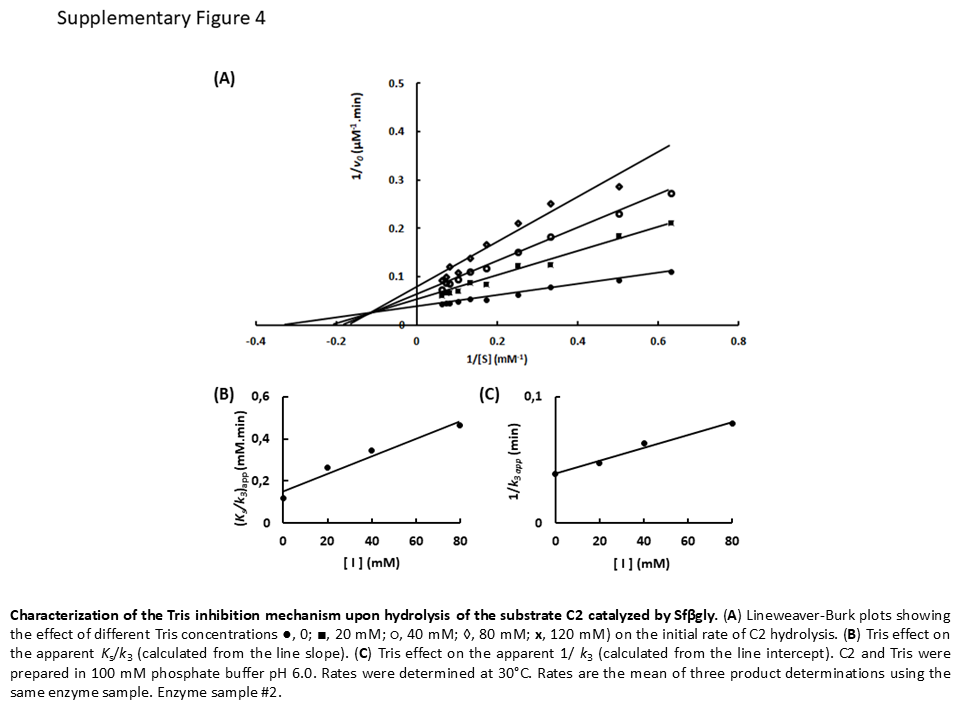

Supplement: S4 Fig — Enzyme sample #2. Substrate C2. (TIF) [file pone.0320120.s004.tif]

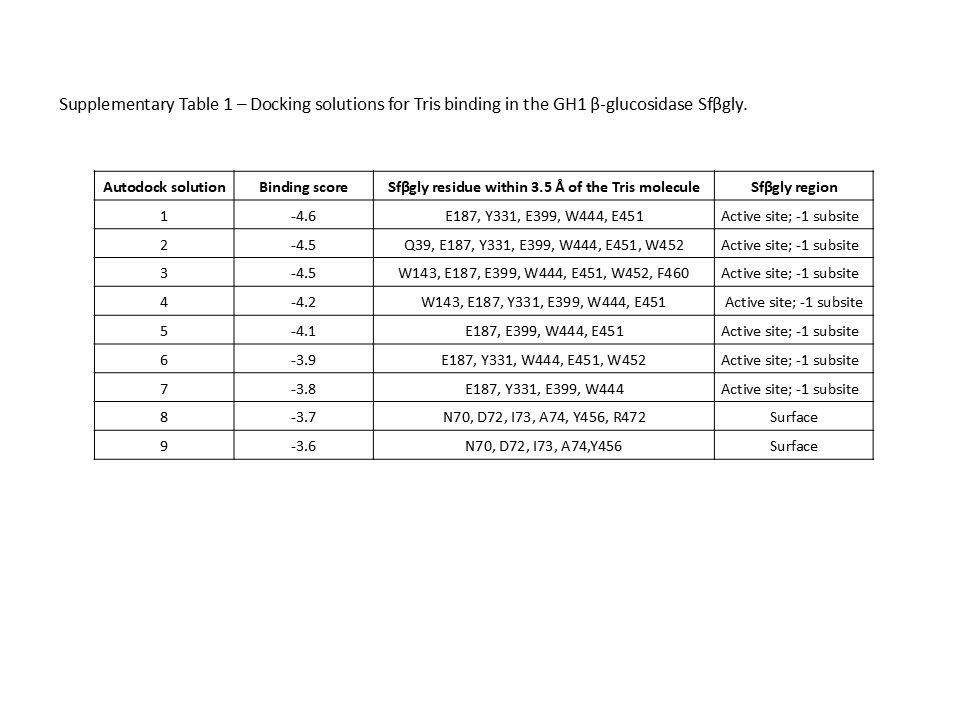

Supplement: S1 Table — (TIF) [file pone.0320120.s005.tif]

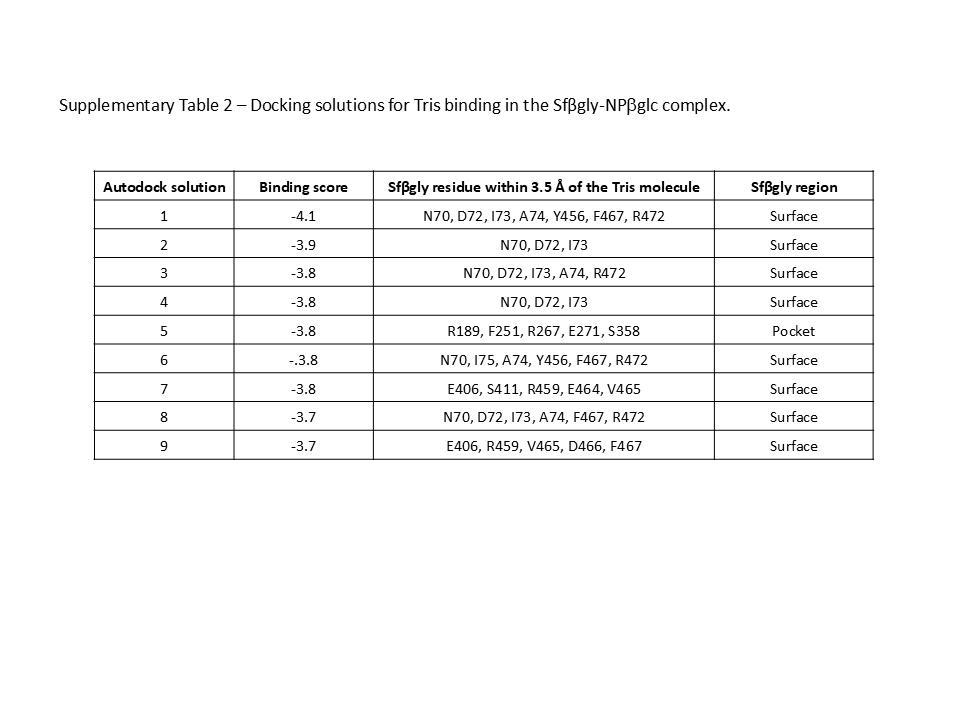

Supplement: S2 Table — (TIF) [file pone.0320120.s006.tif]

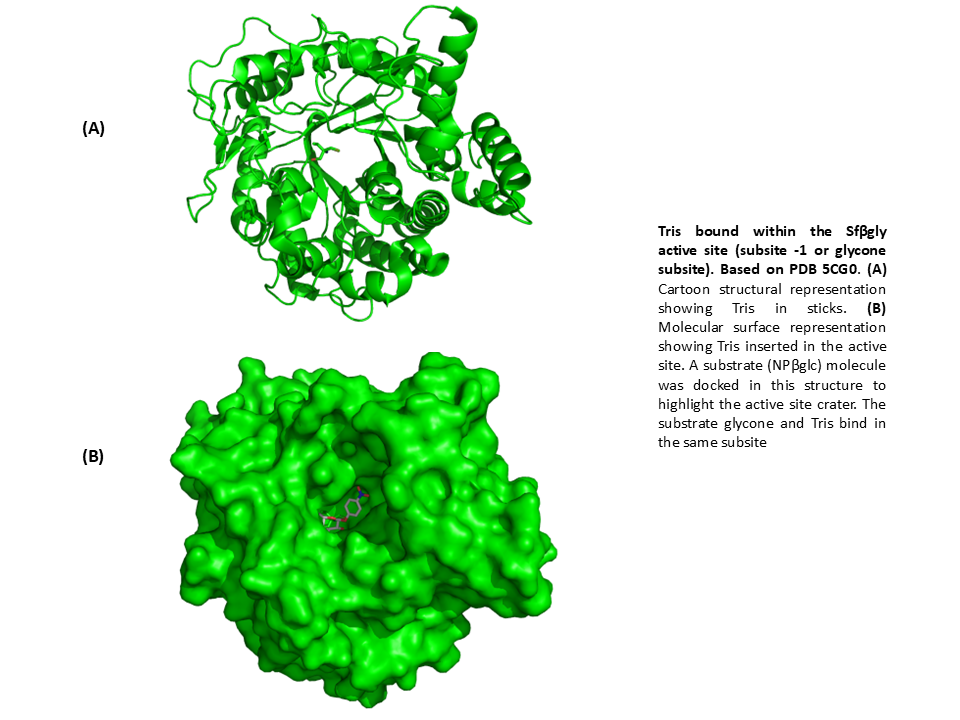

Supplement: S5 Fig — Based on PDB 5 CG0. (TIF) [file pone.0320120.s007.tif]

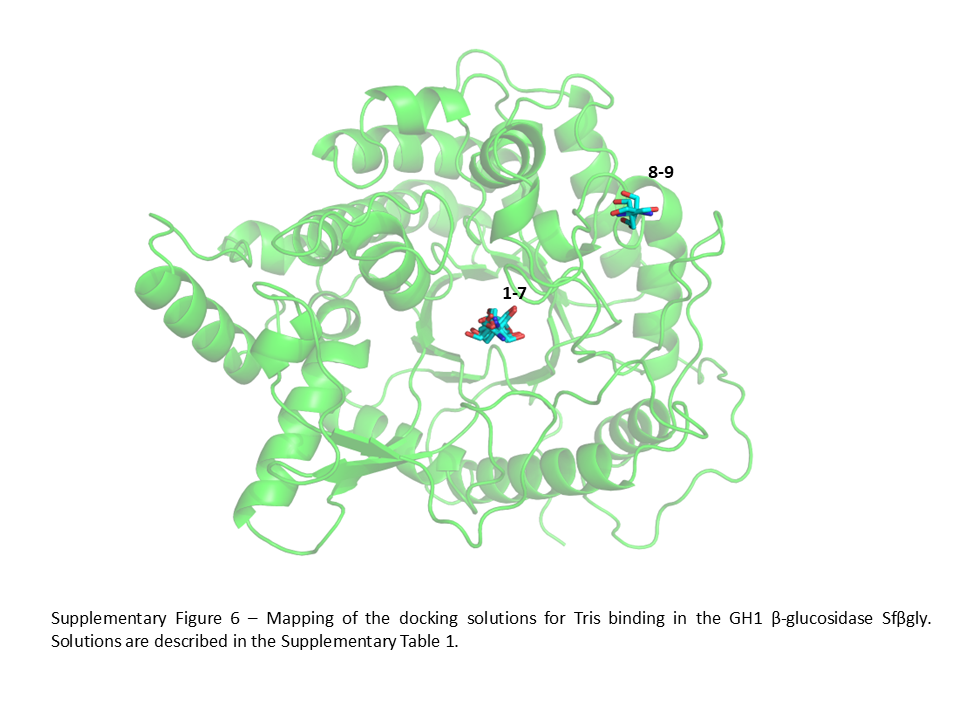

Supplement: S6 Fig — (TIF) [file pone.0320120.s008.tif]

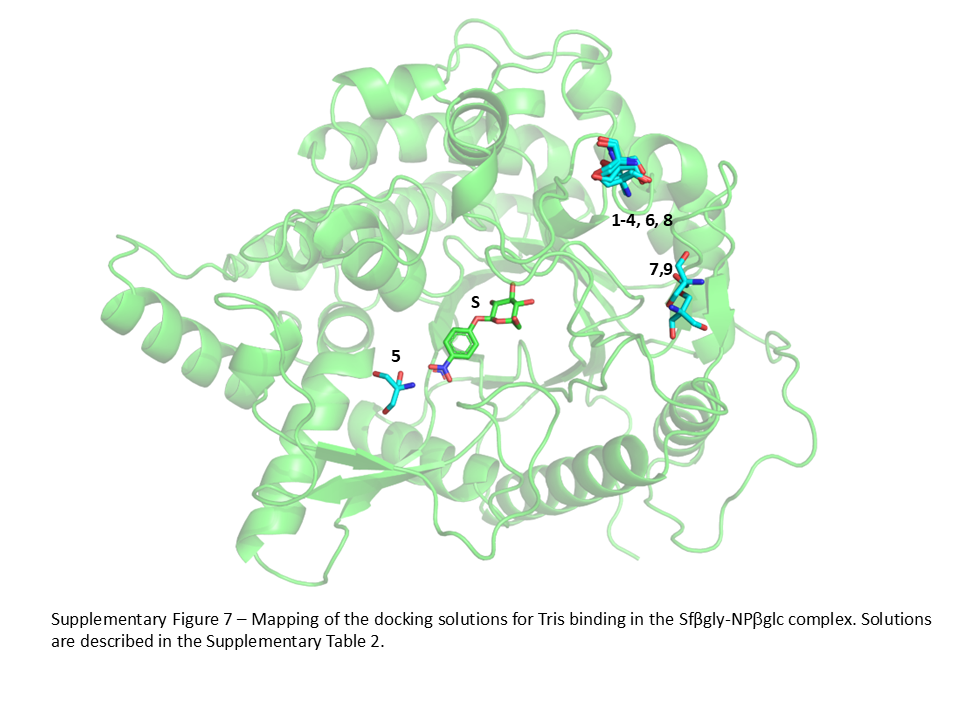

Supplement: S7 Fig — (TIF) [file pone.0320120.s009.tif]

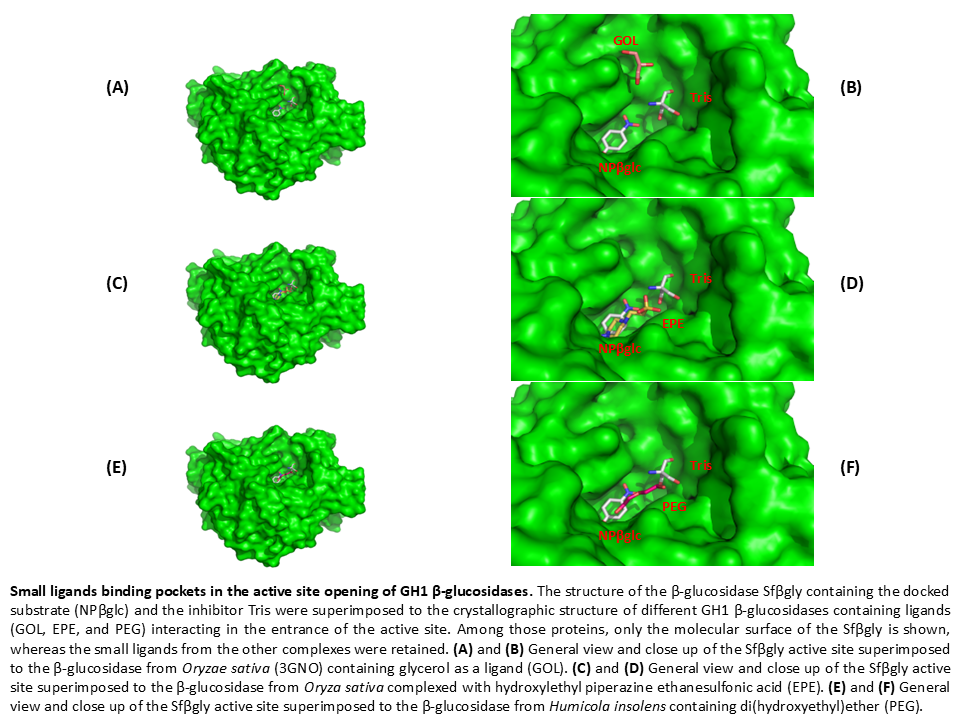

Supplement: S8 Fig — (TIF) [file pone.0320120.s010.tif]

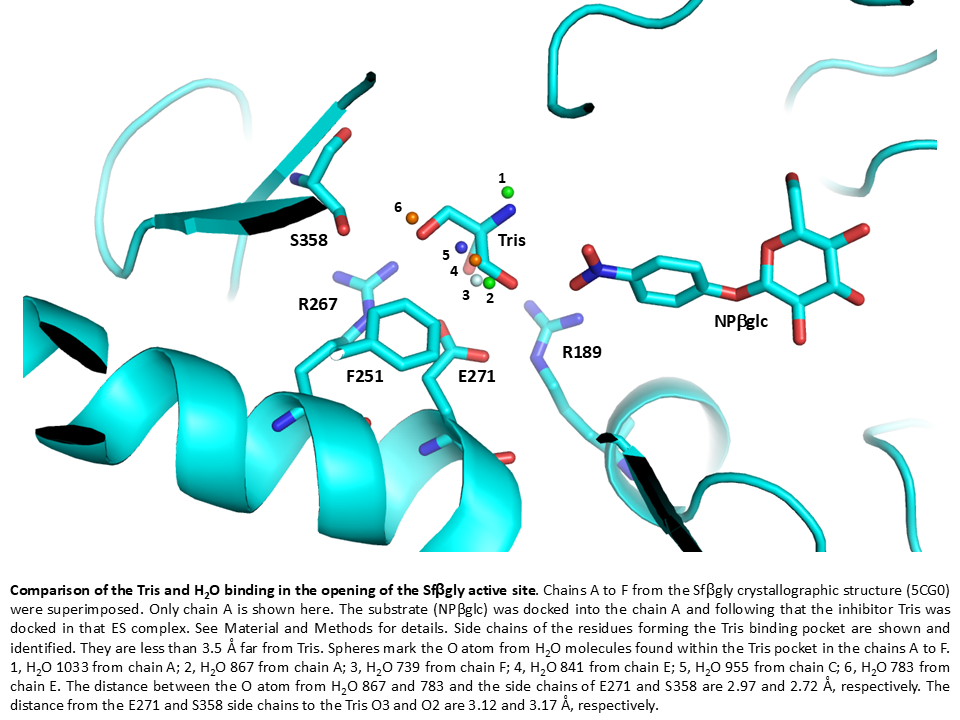

Supplement: S9 Fig — (TIF) [file pone.0320120.s011.tif]

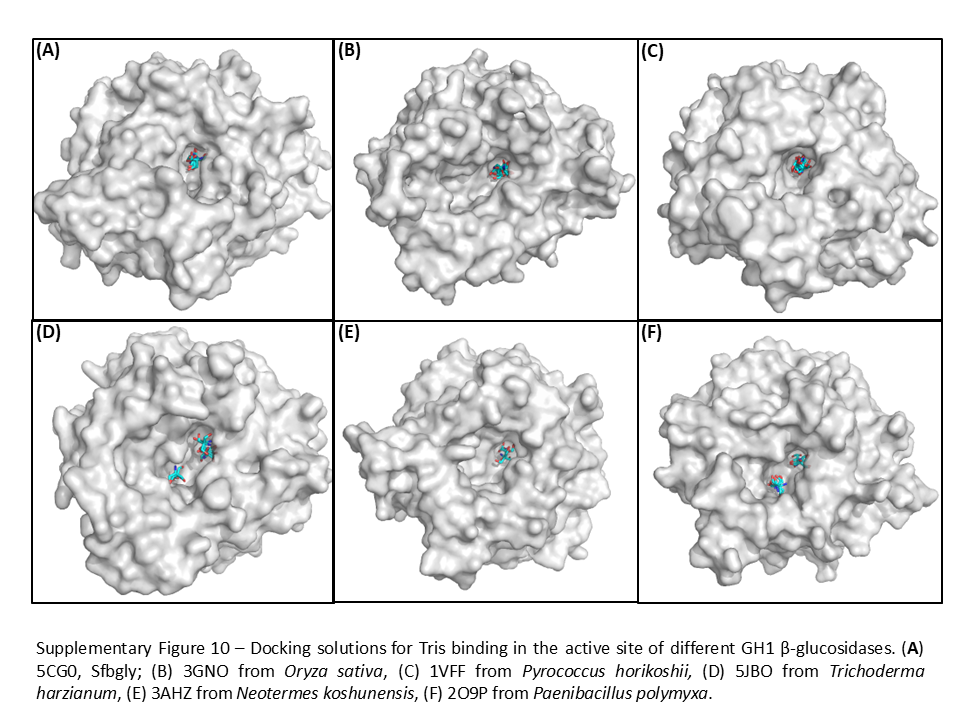

Supplement: S10 Fig — (TIF) [file pone.0320120.s012.tif]
